# Discrimination between protein glycoforms using lectin-functionalised gold nanoparticles as signal enhancers†

**DOI:** 10.1039/d2nh00470d

**Published:** 2023-01-18

**Authors:** Marta M. P. S. Neves, Sarah-Jane Richards, Alexander N. Baker, Marc Walker, Panagiotis G. Georgiou, Matthew I. Gibson

**Affiliations:** a Department of Chemistry, University of Warwick Coventry CV4 7AL UK m.i.gibson@warwick.ac.uk; b Institute of Advanced Study, University of Warwick Coventry CV4 7AL UK; c Department of Physics, University of Warwick Coventry CV4 7AL UK; d Division of Biomedical Sciences, Warwick Medical School, University of Warwick Coventry CV4 7AL UK

## Abstract

Glycoforms (and other post-translational modifications) of otherwise identical proteins can indicate pathogenesis/disease state and hence new tools to detect and sense a protein's glycosylation status are essential. Antibody-based assays against specific protein sequences do not typically discriminate between glycoforms. Here we demonstrate a ‘sandwich’ bio-assay approach, whereby antibodies immobilised onto biolayer interferometry sensors first select proteins, and then the specific glycoform is identified using gold nanoparticles functionalised with lectins which provide signal enhancement. The nanoparticles significantly enhance the signal relative to lectins alone, allowing glycoform specific detection as low as 0.04 μg mL^−1^ (1.4 nM) in buffer, and crucially there is no need for an enrichment step and all steps can be automated. Proof of concept is demonstrated using prostate specific antigen: a biomarker for prostate cancer, where glycoform analysis could distinguish between cancerous and non-cancerous status, rather than only detecting overall protein concentration.

## Abstract 2

Biolayer interferometry is used to distinguish between glycoforms of the same using lectin-functional nanoparticles as both readers, and signal enhancers. The use of this is exemplified with prostate specific antigen.

## Graphical abstract

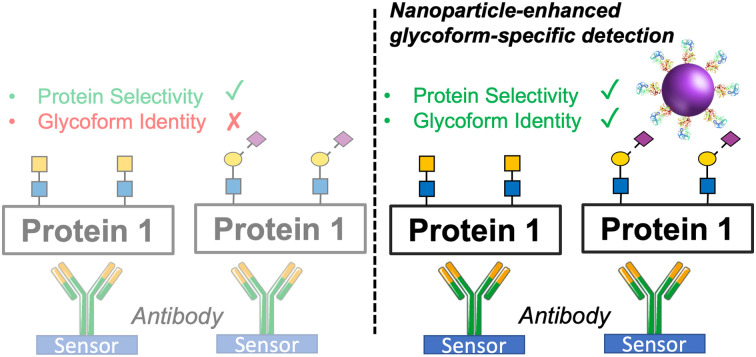

**New concepts**

Here we demonstrate the concept of using biolayer interferometry (BLI) with nanoparticle signal-enhancers to ‘read’ protein glycoforms rapidly and easily, with the works’ impact shown using prostate cancer biomarkers. Protein sequencing and identification tools typically ignore the glycans on the protein: In the case of prostate specific antigen (PSA), this leads to false positives/negatives. The same protein, but with different glycans can indicate healthy tissue or disease. We achieve this by using antibodies on the BLI to first capture ‘all correct protein sequences’ and then using nanoparticles functionalised with lectins capable of recognising a specific glycan. The nanoparticles provide signal enhancement allowing detection at in the nM range, and the entire assay can be automated and complete in under 90 minutes. Current state of the art for glycoform analysis is multistep, laborious mass spectrometry, needing specialised equipment and highly trained researchers. Glycoform discrimination for PSA has been shown in lateral flow, but the detection limits were 50 fold higher than here and required an enrichment step. Our approach is fast and sensitive, bringing the best of both worlds.

## Introduction

Advances in proteomics allows for the routine sequencing of proteins, but this does not routinely capture post-translational modifications (PTMs), including glycosylation, but also lipidation and phosphorylation.^1^ Aberrant (incorrect) glycosylation is a hallmark of various disease states including cancer,^2^ and so it is crucial to be able to identify the exact protein glycoform, not just the sequence, to enable robust diagnostics and biosensing. Glycan analysis can be achieved by degradation/chromatography or by mass spectrometry^3^ but requires multistep sample preparation and/or significant infrastructure and expertise.^4,5^ Lectins are the ‘readers’ of glycosylation *in vivo*,^6^ but have relatively low affinity (mM typically), requiring multivalency^7^ (of lectin and/or glycan) for sensitive detection. Antibodies against glycans can show high affinity but are challenging to generate due to the low immunogenicity of glycans.^8^ Enzyme-derived engineered proteins with antibody like affinity have been demonstrated.^9^ Lectins, in contrast, are easily accessible particularly from plants, and can bind a wide range of glycan structures. Lectins have been reported as the biorecognition elements or detection probes in biosensing of glycosylated cancer biomarkers such as carcinoembryonic antigen (CEA),^10^ alpha-fetoprotein (AFP)^11^ and cancer antigen 125 (CA125).^12^ Lectins have been explored as probes for liquid biopsy of circulating tumour cells for selective capturing and profiling their altered cell–surface glycosylation.^13^ The glycan structure of extracellular vesicles released from different types of cells have also been deciphered using lectin microarrays.^14–16^ Furthermore, lectins are essential tools for basic research where they can be used to assess protein and cell glycosylation structures through a high-throughput lectin barcode patterning^17,18^ as well as applied to biomarker discovery research.^19^ Engineered chimeric antigen receptor (CAR) T cells decorated with lectins were able to target tumour-associated glycosphingolipid globotriaosylceramide (Gb3 – a glycan), demonstrating how glycoform targeting has real biomedical application.^20^

The importance of studying aberrant glycosylation in cancer^21^ is highlighted by the case of the prostate specific antigen (PSA). PSA is a clinical biomarker for prostate cancer, a disease estimated to have led to 375 000 deaths and 1.4 million new cases worldwide in 2020.^22^ Routine PSA testing measures the total protein concentration (not the glycoform) and it is known that higher levels of PSA can also be a sign of other non-malignant conditions,^23^ such as benign prostatic hyperplasia, prostatitis infection, or even can be raised due to patients’ age, previous urologic procedures, medications and vigorous exercise, leading to false positive results.^24^ Changes in the glycosylation patterns on the PSA protein backbone have been correlated with the biomarker being cancer-related or not,^25^ with the specific glycans able to indicate if it is indolent (non-aggressive) or aggressive (and hence needing clinical intervention).^26,27^ Increased levels of *N*,*N*-diacetyllactosamine (LacdiNAc)^28^ and α-2,3 sialic acid in PSA are usually associated with cancer, while α-2,6 sialylated PSA glycoforms are more common in non-cancer patients.^27^ Therefore, consideration of only the proteome, and excluding the glycome, means crucial information on disease status is being lost, and introduces significant false-positives or negatives. Similarly, only using lectins in isolation to probe the glycome would identify one or more of the many similarly glycosylated, but different, proteins.

Impedimetric biosensors using antibody/lectin ‘sandwich’ configuration have been successful within clinically significant concentration ranges.^29–31^ However, these electrochemical biosensors are not currently automated nor high-throughput. ELISA-based assays using antibody/peroxidase dual-functionalised magnetic particles, combined with glycan analysis by lectins immobilised on the ELISA plate were successfully used for fPSA (free PSA) glycoprofiling and detection.^28,32^ These assays, whilst high-throughput, did require independent magnetic capture and enrichment steps, conducted outside of the plate. Lateral flow devices using lectins as the stationary phase, and antibody on the gold were reported, but the limit of detection was higher (2 μg mL^−1^) than required for clinical application (0.004 μg mL^−1^).^33^

Here we demonstrate the concept of a ‘sandwich’ nanoparticle-enhanced, biolayer interferometry (BLI) sensing approach to discriminate between PSA glycoforms in an on-line, easy-to-use platform, which does not require an offline enrichment step. Antibodies are used to first select for the PSA protein backbone, but the signal is generated by application of lectin-functionalised gold nanoparticles, which give large BLI responses. In this proof of concept detection is achieved at 0.04 μg mL^−1^ (nM) concentrations. This shows that nanoparticle-linked BLI glycoform biosensing is feasible and benefits from automation in a simple dip-and-read format.

## Results and discussion

**Fig. 1:**
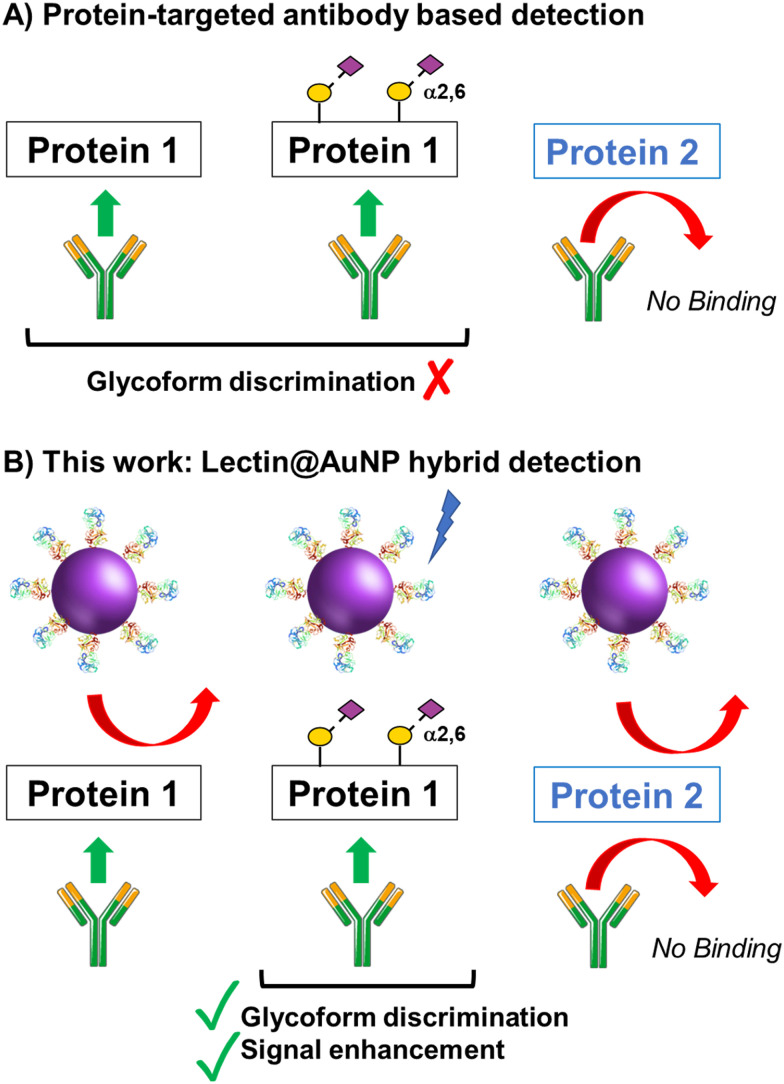
Schematic of (A) single-antibody based detection of glycosylated proteins; (B) Approach taken in this work, using lectin-functionalised nanoparticles to enhance the signal and discrimination of glycoforms captured by a single antibody.

The concept of our hybrid sensing and glycoform discrimination platform is summarised in Fig. 1. A traditional (*e*.*g*. similar to ELISA^34^) based antibody detection platform is shown, whereby two protein sequences can easily be discriminated (Fig. 1A). However, both the glycosylated and non-glycosylated proteins are captured and generate signal, which could be a false positive. Fig. 1B shows our approach where an antibody is used to first capture the correct protein (both glycoforms), which is appealing due to antibodies’ validated selectivity even in complex mixtures. In our approach the signal is only generated following secondary recognition using lectin-coated nanoparticles which can discriminate between glycosylated or non-glycosylated protein. [It should be noted, the aim here is also to discriminate between different glycans, not just binary ‘on/off’]. The sensing output is based on BLI^35^ and hence coupling of lectin to a nanoparticle can lead to an increase in signal compared to using the lectin alone, which has been demonstrated for glycosylated nanoparticles.^36,37^ Previous reports have used immobilised lectin (in lateral flow) followed by antibody-conjugated gold, for PSA glycoform detection.^33^ Our strategy to have a first selection using the antibody is designed to allow extraction of the PSA (in all its glycoforms) before lectin engagement. Due to the potential presence of a large number of other glycosylated proteins in primary samples our sequence of steps is crucial for selectivity.

**Fig. 2:**
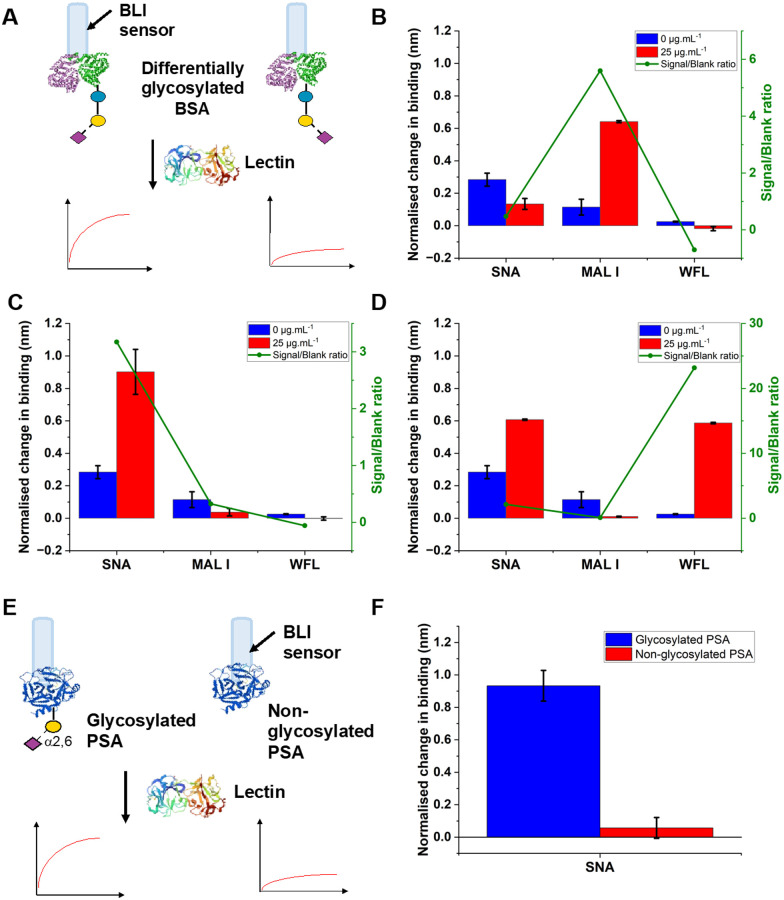
Lectin targeting of glycosylated proteins immobilised on BLI sensors. (A) Schematic of glycosylated bovine serum albumin (25 μg mL^−1^) being interrogated with lectins (30 μg mL^−1^): (B) 2,3′-sialyllactose; (C) 2,6′-sialyllactose; (D) *N*-acetylgalactosamine; (E) Schematic of PSA glycoforms immobilised onto BLI sensor; (F) signal following interrogation with SNA. Error = ± S.D.

To validate the use of BLI and to identify the optimal lectins, a series of model experiments were undertaken. Bovine serum albumin (BSA) glycosylated with 2,3-sialyllactose (2,3′-SL), 2,6,-sialyllactose (2,6′-SL) or *N*-acetyl galactosamine (GalNAc) were immobilised onto carboxylated BLI sensors using EDC/sulfo-NHS coupling (Fig. 2A). Fig. 2B–D shows the BLI response for each BSA glycoform when exposed to a panel of 3 lectins. MAL I (2,3′-SL preference, *Maackia amurensis* lectin I); SNA (2,6′-SL preference, *Sambucus nigra* lectin); WFL (GalNAc preference, *Wisteria floribunda* lectin). In each case the expected binding profiles based on lectin specificities were obtained, validating the use of the lectins to probe glycoforms of proteins immobilised on the BLI sensor. Next, a biomedically relevant protein was used: prostate specific antigen (PSA). PSA is a biomarker in prostate cancer screening/monitoring, but a typical ELISA or lateral flow assay only detects the protein, not the glycoform, which can indicate the difference between healthy and unhealthy PSA. ‘Healthy’ commercial PSA (*i*.*e*., with a high percentage of α-2,6-linked sialic acid) was immobilised onto the BLI sensors in the same manner as BSA, and interrogated with SNA (which prefers 2,6′-SL), Fig. 2E and F. As expected, SNA showed a strong binding affinity to glycosylated PSA compared to non-glycosylated PSA, corroborating again the absence of non-specific binding, consequently SNA was taken forward to the nanoparticle linked assay steps.

**Fig. 3:**
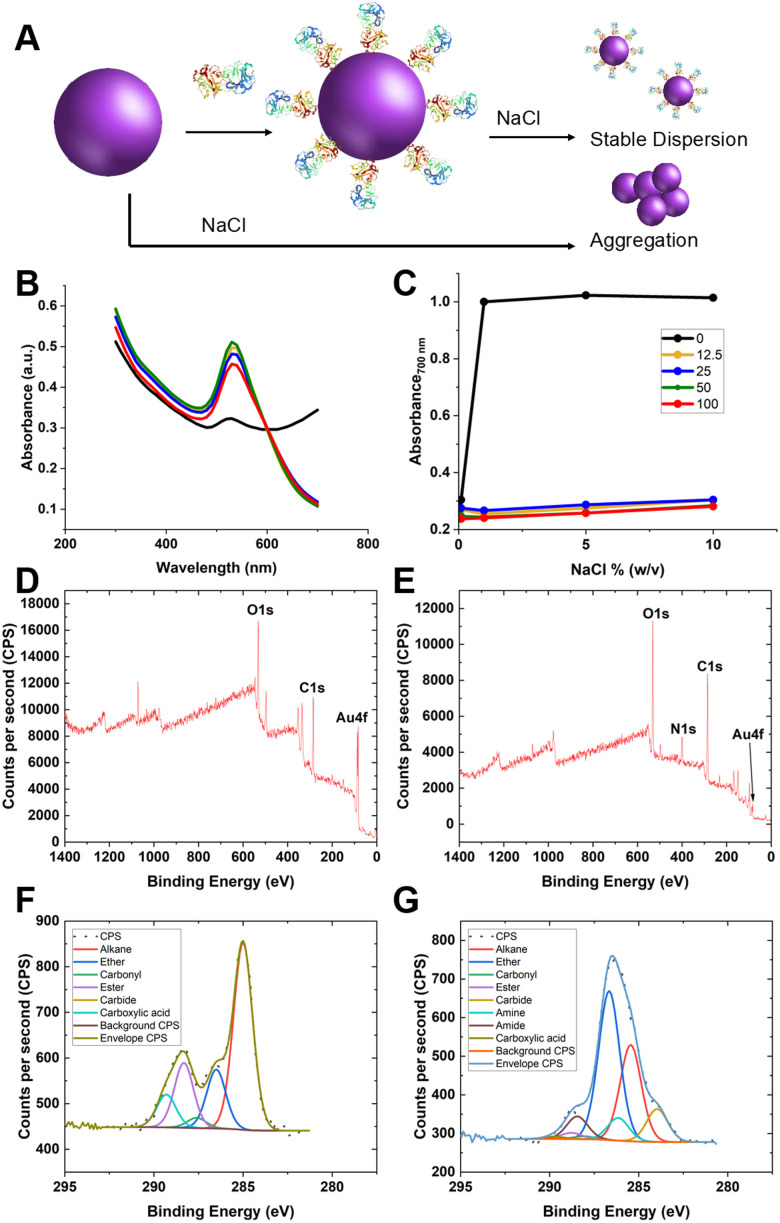
Lectin capture onto gold nanoparticles. (A) Schematic of colloidal stability of lectin coated AuNP *versus* uncoated AuNP, leading to UV-visible changes; (B) example UV-visible spectra for uncoated (black) and coated (others) SNA nanoparticles in 1.7 M NaCl (10% (w/v)); (C) absorbance (700 nm) of nanoparticles with indicated concentration of SNA (μg mL^−1^) applied, as a function of NaCl concentration. Representative XPS survey scan of (D) citrate-AuNP and (E) SNA@AuNP; Representative high-resolution scan of C 1s region (F) citrate-AuNP and (G) SNA@AuNP.

Guided by the above, 35 nm gold nanoparticles were synthesised (see Fig. S2/4 for dynamic light scattering (DLS)/transmission electron microscopy (TEM) characterisation, ESI†) and functionalised with SNA (as the optimal lectin for PSA-glycosylation detection). SNA was absorbed to the surface of citrate-coated gold nanoparticles (AuNPs) providing stabilisation against aggregation, compared to the naked AuNPs alone (Fig. 3A/B). Fig. 3C shows the change in absorbance at 700 nm of 35 nm citrate-stabilised AuNPs at different NaCl concentrations. With no SNA added, the particles aggregated readily, whereas after addition of SNA (12.5–100 μg mL^−1^) the particles did not aggregate, as shown by their red colouration, confirming successful conjugation of the SNA to the particle surface. To further confirm the presence of SNA on the nanoparticle surface X-ray photoelectron spectroscopy (XPS) was used. Fig. 3D and E shows survey scans highlighting the N 1s signal after lectin addition not found in the background. Fig. 3F and G show high-resolution scans of the C 1s region, and incorporation of amide bonds (from protein) post-lectin addition. Further high-resolution scans are including in the ESI† (Tables S1 and S2; Fig. S3). DLS of AuNPs functionalised with lectins also showed an increase in hydrodynamic diameter, which suggests protein coating of the particles and further confirming colloidal stability (Fig. S4, ESI†).

The glycan binding capacity of SNA@AuNP was validated against fetuin (a sialylated protein) as a model glycoprotein. Protein loading was first optimised (Fig. S5, ESI†) and the inter- and intra-batch reproducibility (Fig. S6, ESI†) of the binding outputs were validated as being highly reproducible. The long-term stability of the particles was also shown, retaining function up to week 10 when stored at 4 °C, demonstrating the surface immobilisation leads to stable and functional colloids suitable for sensing applications (see Fig. S7 in ESI†).

**Fig. 4:**
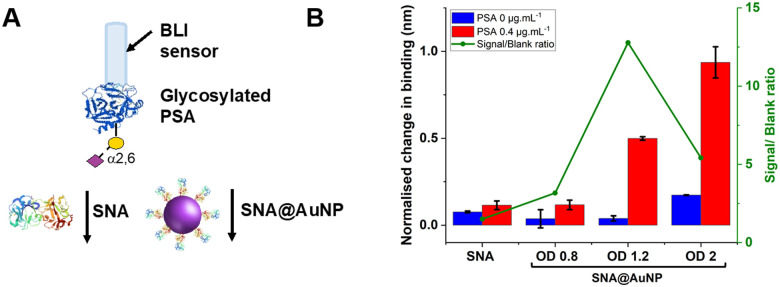
(A) Schematic comparison of lectin *vs*. lectin@AuNP conjugates BLI response in the (B) presence of PSA (0.4 μg mL^−1^) and absence of PSA (negative control). Other experimental conditions: SNA 50 μg mL^−1^; SNA@AuNP (coated with SNA 50 μg mL^−1^). Error = ± S.D.

With these validated and stable lectin-functional nanoparticles to hand, the signal enhancement effect of the SNA@AuNP was tested against glycosylated PSA, Fig. 4A. Glycosylated PSA was captured to a BLI sensor using EDC/sulfo-NHS coupling. Interrogation of the PSA with SNA lectin alone gave small signals but could discriminate between no PSA and 0.4 μg mL^−1^ PSA, Fig. 4B. In contrast when increasing concentrations of SNA@AuNP were added, the discrimination between PSA or no PSA was far stronger giving a maximum signal to noise of 12.8, compared to 1.50 for the lectin alone (Fig. 4B). Negative controls using other lectin-coated particles confirmed that SNA@AuNP was selectively binding the glycans, and ruled out non-specific interactions from protein-coated particles (Fig. S8, ESI†).

**Fig. 5:**
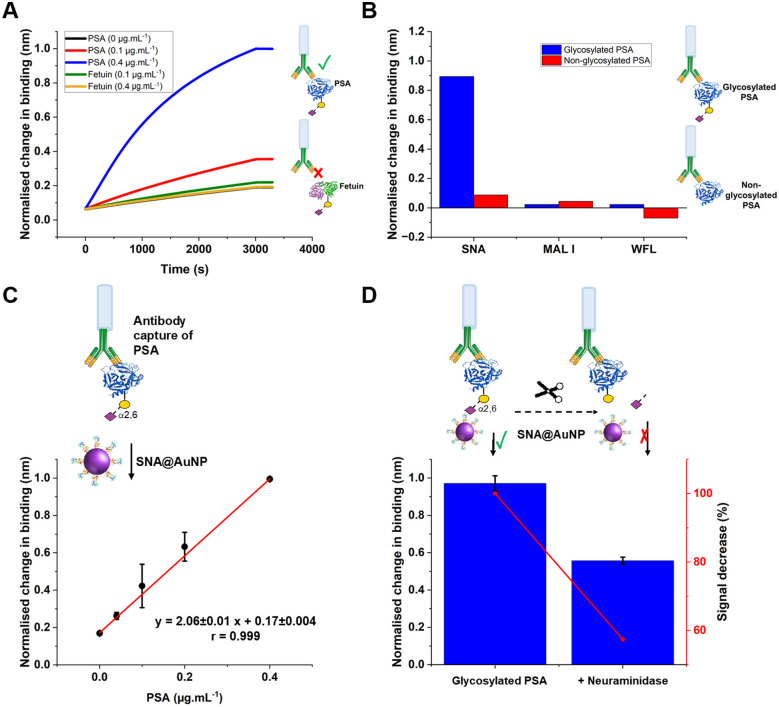
(A) Evaluation of capture antibody specificity for PSA as target analyte in the presence of fetuin (another sialylated glycoprotein recognised by SNA lectin), followed by detection with SNA@AuNP conjugates. (B) Study of non-specific binding of SNA, MAL I and WFL (20 μg mL^−1^) towards different PSA (10 μg mL^−1^) glycoforms. (C) Dose-response *versus* PSA gradient (D) Evaluation of specificity towards PSA (0.4 μg mL^−1^) binding before and after partial enzymatic removal of terminal sialic acid residues after overnight incubation with α2-3,6,8,9 neuraminidase A (120 units mL^−1^). Other experimental conditions: Capture antibody (15 μg mL^−1^ (A, C, and D); 30 μg mL^−1^ (B); SNA@AuNP (coated with SNA 50 μg mL^−1^; OD 1.2).Error = ± S.D.

With the concept of glycoform discrimination confirmed, a more advanced (and relevant) assay was developed, using the complete ‘sandwich’ of antibody on BLI probe (optimisation in Fig. S9, ESI†) and lectin@AuNP. The anti-PSA specific antibody was confirmed to selectively capture the analyte of interest (PSA) compared to fetuin (a control glycoprotein) as shown in Fig. 5A. Lectin binding affinity ensured the correct glycoform identification for identical proteins with different glycan patterns (Fig. 5B). Fig. 5C shows a complete detection system for PSA in the concentration range of 0–0.4 μg mL^−1^. This AuNP linked-BLI system could detect PSA at concentrations as low as 0.04 μg mL^−1^ (1.4 nM), which is above the clinical diagnosis limit (which fails to differentiate between glycoforms) but significantly better than a lateral flow based technique for glycoform analysis which could detect to 2 μg mL^−1^, so a 50-fold improvement.^33^ The clinical utility of glycoform identification at higher PSA levels may be of importance as a tool to avoid invasive biopsies and perform risk stratification.^24^ A glycoform detection tool such as this, could also be deployed following a standard (with lower sensitivity limits) protein-detection tool.

As a further test, glycosylated human PSA was subjected to neuraminidase treatment to selectively cleave some of the terminal sialic acids on the glycoprotein. Note, these enzymes were not 100% efficient and hence this produces a mixture of glycoforms, providing a useful test of the technology. Compared to the fresh glycosylated PSA, the neuraminidase treated sample, showed a 50% reduction in signal, consistent with differentiation between the glycoforms, despite using the same antibody as the primary ‘capture’ unit, Fig. 5D. This shows that our BLI/AuNP linked approach not only provides signal enhancement but that the signal is linked to the extent of the specific glycoform, which is crucial for future glycoform based assays/diagnostics.

A preliminary set of experiments was undertaken using PSA spiked into serum to demonstrate the future potential, and challenges, of this sensing approach in complex media with matrix effects. The results (Fig. S10 in ESI†) did show background interference, but dilution of the media reduced this. We hypothesise that optimisation of the antibody-selection step, and the washing protocol will enable this to be overcome but matrix effects, as with any sensing tool, is a key challenge for real application.

The key advantage of the proposed approach is that it can be automated, without separate enrichment steps and it provides quantitative outputs, unlike lateral flow (which is low cost and easy to use) but gives (typically) qualitative (yes/no) outcomes. Future work will extend to a range of glycoforms associated with different disease states and to reduce matrix effects to build on the proof of concept shown here.

## Conclusions

Here we have demonstrated that sensitive detection and discrimination of protein glycoforms can be achieved by combining biolayer interferometry with signal enhancement from lectin-functional gold nanoparticles. Antibodies immobilised on the BLI sensors allow capture of proteins based on their sequence, and subsequent interrogation with lectin@AuNPs that ‘read’ the glycan enabling selective identification of the presence of a particular glycoform. This method is shown to be more sensitive than using lectins alone. The system was optimised for glycoform detection using glycoproteins and a panel of lectins. As a model, but clinically relevant, detection scenario prostate specific antigen (PSA) was used. PSA is a key prostate cancer biomarker, but different glycoforms are associated with false negative/positive diagnostics. SNA lectin was identified as being able to detect PSA glycosylation and was immobilised onto gold nanoparticles. Crucially the saline stability and the reproducibility of the gold coating was demonstrated. Using this system, it was possible to detect a specific glycoform of PSA down to 0.04 μg mL^−1^ (1.4 nM). Our results demonstrate that by coupling lectins to ‘read’ the glycosylation status of proteins, combined with established antibody-based purification/isolation, in a nanoparticle enhanced BLI platform, it is possible to rapidly obtain glycoform information. BLI is high-throughput (using multiwell plates), can be easily automated and provides quantitative data outputs. The automation may also facilitate technology transfer to other setting by reducing training/skills needed to operate. This approach is complementary to low-cost methods such as lateral flow or conventional ELISA type sensing paradigms and could be applied to many other glycoforms-reading challenges. The concept could potentially be applied to other post-translational modifications, subject to appropriately functionalised particles being available.

## Author contributions

M.M.P.S.N. conducted all BLI assays and nanoparticle conjugation. S.J.R. carried out nanoparticle synthesis and DLS experiments. A.N.B. and M.W. undertook XPS analysis. P.G.G. carried out TEM analysis. M.I.G. oversaw the project and directed the research. M.M.P.S.N. and M.I.G. wrote the paper and all authors contributed and commented on the manuscript.

## Conflicts of interest

There are no conflicts to declare.

## Supplementary Material

NH-008-D2NH00470D-s001
