# Supplementary material for: Discrimination between protein glycoforms using lectin-functionalised gold nanoparticles as signal enhancers
Source: Nanoscale Horiz. 2023 Jan 18;8(3):377–82. doi: 10.1039/d2nh00470d (PMC9969229; doi:10.1039/d2nh00470d)
Supplement: NH-008-D2NH00470D-s001 [file NH-008-D2NH00470D-s001.pdf]

Electronic Supplementary Material (ESI) for Nanoscale Horizons.
This journal is © The Royal Society of Chemistry 2023

Supporting information for

Discrimination between protein glycoforms using lectin-functionalised gold nanoparticles as signal enhancers

Marta M. P. S. Neves,[a,b] Sarah-Jane Richards,[a] Alexander N. Baker[a], Marc Walker,[c] Panagiotis G. Georgiou[a], and Matthew I. Gibson*[a,d]

Author affiliations:

[a]Department of Chemistry, University of Warwick, CV4 7AL, UK

[b]Institute of Advanced Study, University of Warwick, CV4 7AL, UK

[c]Department of Physics, University of Warwick, Coventry, CV4 7AL, UK

[d]Division of Biomedical Sciences, Warwick Medical School, University of Warwick, CV4 7AL, UK

Corresponding Author Email: m.i.gibson@warwick.ac.uk

Contents

Contents..S2

Experimental...S3

Reagents and solutions………...S3

Gold nanoparticles (AuNPs) synthesis……………..S3

AuNPs functionalisation with lectins…………………………..............................S4

Dynamic Light Scattering (DLS)..S4

Transmission Electron Microscopy (TEM)…...S4

X-ray Photoelectron Spectroscopy (XPS) S4

UV-vis Spectroscopy...S5

Biolayer Interferometry (BLI)..S5

Serum samples analysis………………………………………………………………S6

Additional Data and Figures...S7

References...S18

Experimental

Reagents and solutions

Sodium citrate tribasic dihydrate (> 99 %), gold(III) chloride trihydrate (99.9%), HEPES, sodium chloride (NaCl), calcium chloride ($CaCl_2$), sodium hydroxide (NaOH), ethanolamine hydrochloride, *N*-(3-dimethylaminopropyl)-*N′*-ethylcarbodiimide hydrochloride (EDC), *N*-hydroxysulfosuccinimide sodium salt (sulfo-NHS), and heat inactivated, sterile-filtered human serum, from human male AB plasma, were obtained from Sigma-Aldrich. Sodium acetate trihydrate, acetic acid (glacial), magnesium chloride ($MgCl_2$) were provided from Fisher Scientific. *Sambucus Nigra* Lectin (SNA), *Maackia Amurensis* Lectin I (MAL I) and *Wisteria Floribunda* Lectin (WFL) were acquired from Vector Laboratories. 3'-sialyllactose (3 atom spacer), 6'-sialyllactose (3 atom spacer) and *N*-acetylgalactosamine-BSA were purchased from Dextra Laboratories and α2-3,6,8,9 Neuraminidase A sialidase (20,000 $U.mL^{-1}$) from New England Biolabs. Mouse monoclonal [8301] to Prostate Specific Antigen (anti-PSA Ab) and Native Human Prostate Specific Antigen (PSA) protein were supplied by Abcam. Working solutions of anti-PSA Ab, PSA and AuNPs@lectins were prepared in 10 mM HEPES buffer pH 7.5 with 100 mM NaCl, 0.1 mM $CaCl_2$, 0.01 mM $MgCl_2$. EDC/sulfo-NHS solution was freshly prepared every day, before the experiment begins, in acetate buffer pH 5.0. Ultrapure water (resistivity =18.2MΩ.cm (at 25 °C)) was used throughout this work. All chemicals employed were of analytical reagent grade.

Gold nanoparticles (AuNPs) synthesis

35 nm gold nanoparticles (AuNPs) were synthesised following a method described elsewhere.[1] Briefly, 150 mL of a 2.2 mM sodium citrate solution was heated under reflux for 15 min under vigorous stirring. Then, 1 mL of $HAuCl_4$ (25 mM) was injected once the solution started boiling. A change of colour from yellow to bluish grey and finally to soft pink was observed within a 10 min period indicating the formation of the gold seeds. Afterwards, the solution was cooled down until the temperature reached 90 °C, when 1 mL of $HAuCl_4$ solution (25 mM) was again injected. After 20 min, the reaction was finished, and this process was repeated twice. Afterwards, the sample was diluted by adding 85 mL of MilliQ water and 3.1 mL of 60 mM sodium citrate. This solution was then used as a seed solution, and three further portions of 1.6 mL of 25 mM $HAuCl_4$ were added with 20 min between each addition. Following completion

of this step 1 mL was taken for DLS and UV/Vis analysis. The sample was diluted by adding 135 mL of MilliQ water and 4.9 mL of 60 mM sodium citrate. This solution was then used as a seed solution, and the process was repeated with three further additions of 2.5 mL of 25 mM $HAuCl_4$, this solution was analysed by DLS and UV/Vis and target size of 35 nm was reached and the solution was cooled and a sample taken for TEM analysis.

AuNPs functionalisation with lectins

The AuNPs were coated with the lectins SNA, MAL I, WFL (final concentration 50 µg.mL^{-1}) in a reproducible and stable manner for 7 hours, with gentle stirring at room temperature. After a centrifugation step (4500 rpm) for 20 min the supernatant was removed, eliminating the unbound protein, and the Lectin@AuNP conjugates pellet was re-dispersed in 850 µL of HEPES buffer pH 7.5.

Dynamic Light Scattering (DLS)

Hydrodynamic diameters (Dh) and size distributions of particles were determined by DLS using a Malvern Zetasizer Nano ZS with a 4 mW He-Ne 633 nm laser module operating at 25 °C. Measurements were carried out at an angle of 173° (back scattering), and results were analysed using Malvern DTS 7.03 software. All determinations were repeated 5 times with at least 10 measurements recorded for each run. Dh values were calculated using the Stokes-Einstein equation where particles are assumed to be spherical.

Transmission Electron Microscopy (TEM)

Dry-state stained TEM imaging was performed on a JEOL JEM-2100Plus microscope operating at an acceleration voltage of 200 kV. All dry-state samples were diluted with deionised water and then deposited onto formvar-coated copper grids.

X-Ray Photoelectron Spectroscopy (XPS)

The samples were attached to electrically-conductive carbon tape, mounted on to a sample bar and loaded in to a Kratos Axis Ultra DLD spectrometer which possesses a base pressure below 1 x 10^{-10} mbar. XPS measurements were performed in the main analysis chamber, with the sample being illuminated using a monochromated Al Kα x-ray source. The measurements were

conducted at room temperature and at a take-off angle of 90° with respect to the surface parallel. The core level spectra were recorded using a pass energy of 20 eV (resolution approx. 0.4 eV), from an analysis area of 300 μm x 700 μm. The spectrometer work function and binding energy scale of the spectrometer were calibrated using the Fermi edge and $3d_{5/2}$ peak recorded from a polycrystalline Ag sample prior to the commencement of the experiments. In order to prevent surface charging the surface was flooded with a beam of low energy electrons throughout the experiment and this necessitated recalibration of the binding energy scale. To achieve this, the C-C/C-H component of the C 1*s* spectrum was referenced to 285.0 eV. The data were analysed in the CasaXPS package, using Shirley backgrounds and mixed Gaussian-Lorentzian (Voigt) lineshapes. For compositional analysis, the analyser transmission function has been determined using clean metallic foils to determine the detection efficiency across the full binding energy range.

UV-vis Spectroscopy

Absorbance measurements were recorded on a BioTek Epoch Microplate Reader using 96-well plates purchased from Greiner Bio-one.

Biolayer Interferometry (BLI)

Biolayer Interferometry was carried out on ForteBio Octet Red96 (Forte Bio, USA) instrument (software version 9.0) and amine reactive (ARG2) biosensor tips (Forte Bio, USA) were used. All bioassays were performed in black 96-well plates acquired from Greiner Bio-one. The optimised BLI-bioassay (illustrated in Figure S1) was setup and preformed according to the following procedure: Amine reactive (ARG2) biosensor tips, previously hydrated in milliQ H2O water were (1) dipped in milliQ water for 1 minute to ensure that a stable baseline was established. Afterwards, the biosensors were first activated using (2) EDC/sulfo-NHS for 5 minutes and functionalised by (3) loading with 15 $\mu g.mL^{-1}$ anti-PSA antibody in acetate buffer pH 5 for 5 mins followed by (4) 5 minutes quenching with 1 M ethanolamine in PBS pH 8.0 and (5) 2 mins washing step in 10 mM HEPES buffer pH 7.5 with 100 mM NaCl, 0.1 mM $CaCl_2$, 0.01 mM $MgCl_2$ to remove any unbound protein and to establish a stable baseline. Following the capture antibody immobilisation, the (6) first binding step with PSA (or human serum spiked with PSA) was carried out in 10 mM HEPES buffer pH 7.5 with 100 mM NaCl,

0.1 mM $CaCl_2$, 0.01 mM $MgCl_2$ for 10 minutes followed by (7) dissociation in 10 mM HEPES buffer pH 7.5 with 100 mM NaCl; 0.1 mM $CaCl_2$, 0.01 mM $MgCl_2$ for 2 minutes to again to remove any non-specific protein. Finally, the detection step was carried out in the (8) second binding association event (50 min) with lectin-functionalised gold nanoparticles (Lectin@AuNP). SNA@AuNP were used to detect glycan profiles not related to prostate cancer whereas AuNPs conjugated to MAL I and WFL, specific to cancer-related PSA glycoforms, where tested as negative controls. A last dissociation step in 10 mM HEPES buffer pH 7.5 with 100 mM NaCl, 0.1 mM $CaCl_2$, 0.01 mM $MgCl_2$ for 5 minutes finalised the experiment. All bioassays were performed at 30 °C and agitated at 1,000 rpm. The signal enhancement (wavelength shift, Δλ) observed in the second association step was correlated with PSA presence, concentration, and glycan profile.

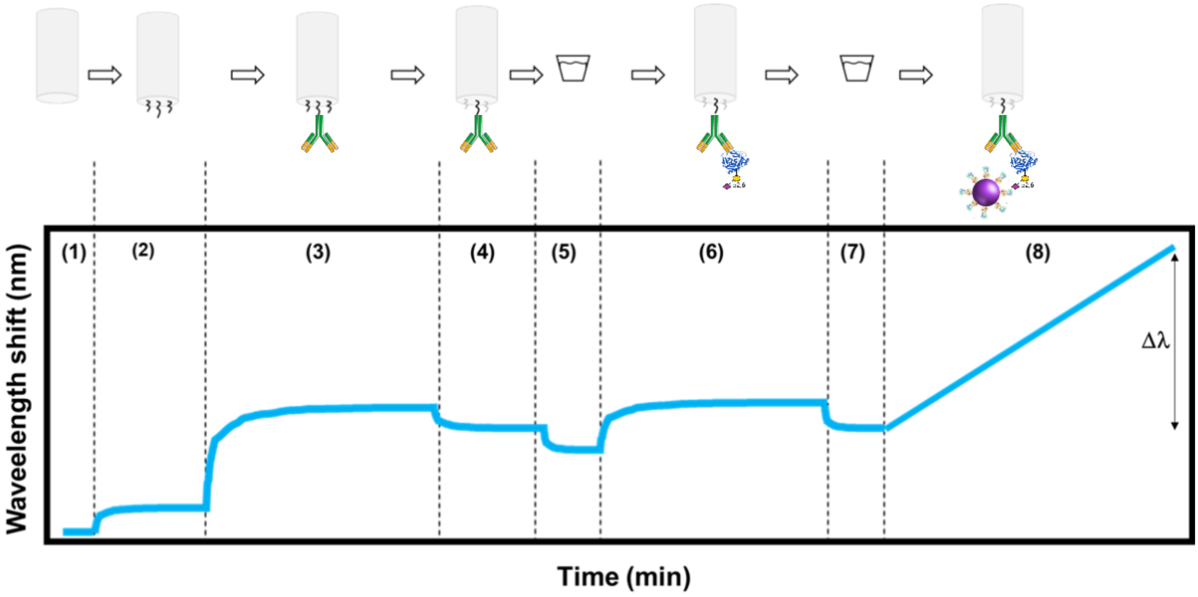

Figure S1. Schematic representation of BLI assay.

Serum samples analysis

Serum samples diluted (1:10; 1:100; 1:1000; 1:2000; 1:5000) in 10 mM HEPES buffer pH 7.5 with 100 mM NaCl, 0.1 mM $CaCl_2$, 0.01 mM $MgCl_2$ were spiked with PSA 0.1 μg.mL^{-1}. The measurements obtained following the protocol described in the previous section (Biolayer Interferometry (BLI)) were compared to the signal obtained for PSA 0.1 μg.mL^{-1} in buffer (control solution). Unspiked serum samples and buffer solution (i.e. PSA 0 μg.mL^{-1}) were used as additional controls.

Additional Data and Figures

AuNPs characterisation

The citrate-stabilised gold nanoparticles were also characterised by DLS and TEM analysis. Example images and histogram analyses of colloidally stable particles are shown below.

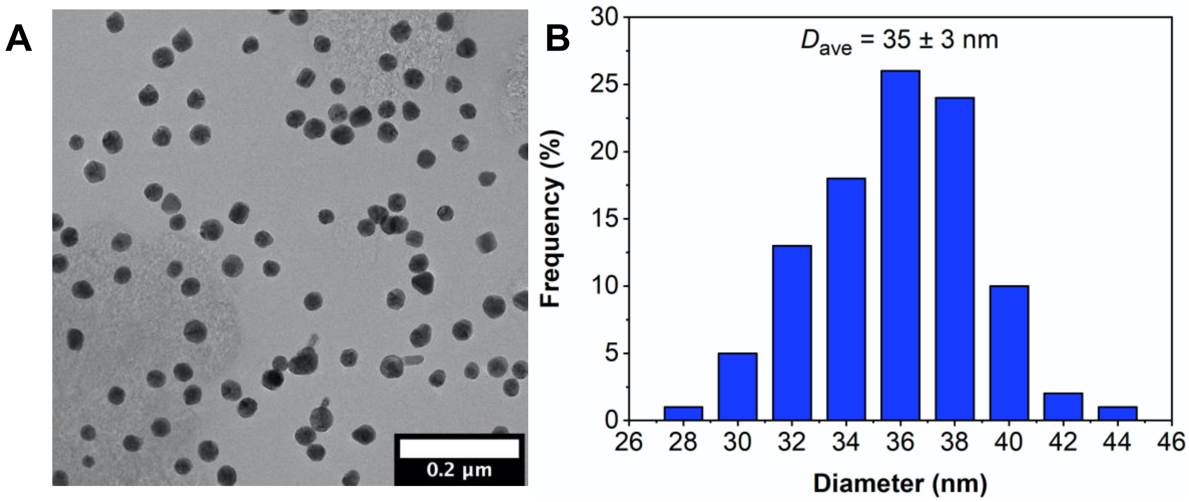

Figure S2. TEM images (A) and histograms (B) of citrate stabilised 35 nm AuNPs. Histograms from analysis of >100 particles.

Analysed XPS data

Lectin coating was further confirmed by use of XPS. In particular, the presence of amides [from N 1*s*], that are not present on the naked particles, confirmed successful binding of the lectins onto the particle surface.

Table S1. Elemental compositions of particles from XPS

**Particle**	**Elemental Percentage Composition (%)**				**Elemental ratios**	
	C 1*s*	O 1*s*	N 1*s*	Au 4*f*	N 1*s*/ C 1*s*	N 1*s*/ Au 4*f*
**Uncoated AuNP 35 nm_batch 1**	56.15	33.68	0.67	9.51	0.01	0.07
**Uncoated AuNP 35nm_batch 2**	57.36	37.12	0.38	5.14	0.01	0.07
**Lectin-coated AuNP 35nm_batch 1**	57.53	31.42	10	1.05	0.17	9.52
**Lectin-coated AuNP 35nm_batch 2**	58.38	34.31	7.09	0.23	0.12	30.83

Table S2. C 1*s* Bond Percentage Composition (%)

Particle	C 1*s* Bond Percentage Composition (%)								Bond Ratios	
	Alkane	Ether	Carbonyl	Ester	Carbide	Amine	Amide	Carboxylic acid	Amide/Alkane	Amide/Ether
**Uncoated AuNP 35nm_batch 1**	52.76	16.65	2.91	18.33	0.08	0	0	9.26	0.00	0.00
**Uncoated AuNP 35nm_batch 2**	39.25	21.77	6.57	21.55	0	0	0	10.86	0.00	0.00
**Lectin-coated AuNP 35nm_batch 1**	28.76	44.45	0.87	1.86	9.81	6.78	6.78	0.68	0.24	0.15
**Lectin-coated AuNP 35nm_batch 2**	29.81	37.60	6.58	0	11.04	5.78	5.78	3.40	0.19	0.15

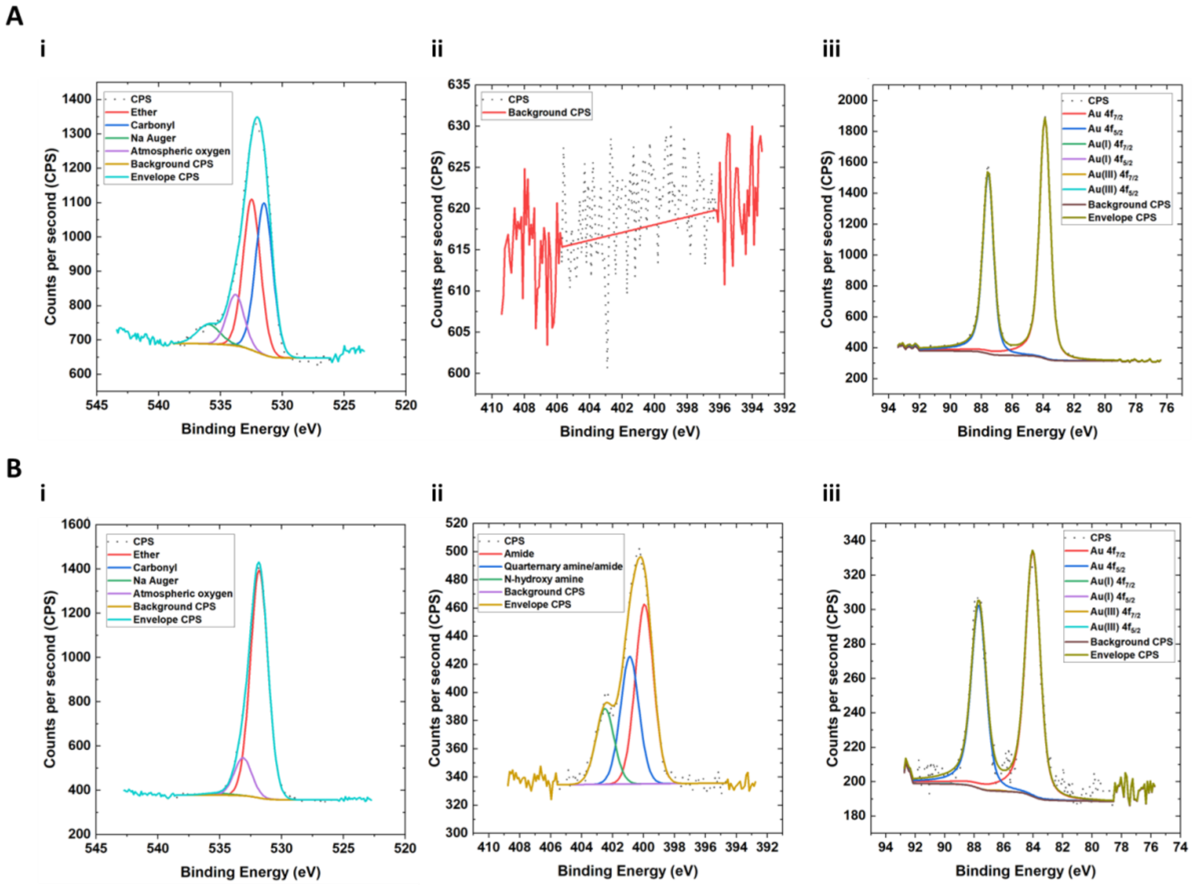

Figure S3. Representative XPS of (A) uncoated AuNP and (B) lectin coated AuNP (SNA@AuNP): (i) O 1*s* (ii) N 1*s* (iii) Au 4*f*.

DLS analysis of uncoated and lectin-coated AuNPs

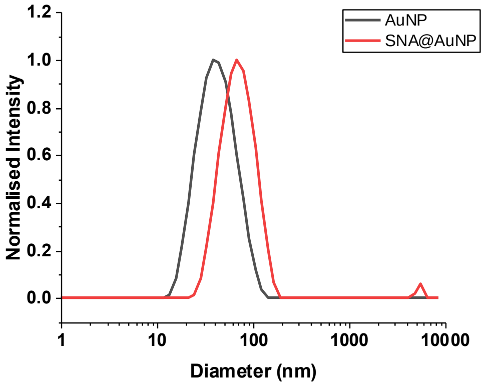

Figure S4. Hydrodynamic diameter (from DLS) of uncoated 35 nm AuNPs (black) and SNA-functionalised 35 nm AuNPs (red).

Optimisation of the lectin concentration used to functionalise gold nanoparticles

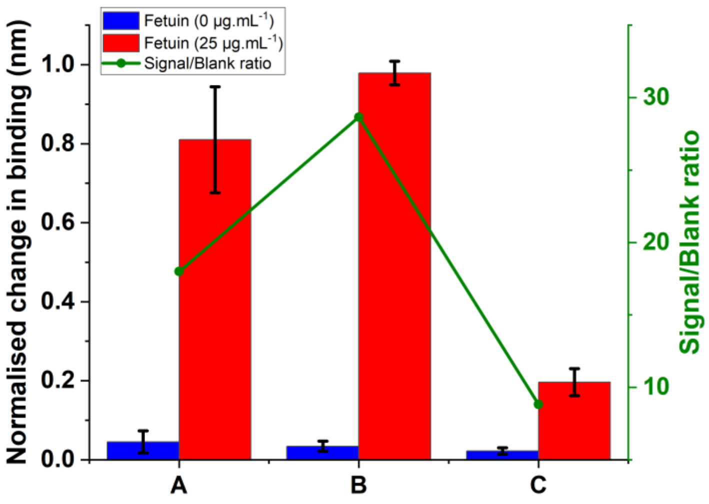

Figure S5. Optimisation of AuNP lectin coating with SNA ((A) 25 µg.mL^{-1}, (B) 50 µg.mL^{-1}) and comparison with (C) SNA (50 µg.mL^{-1}) performance as control. The optimum amount of lectin coating was evaluated in terms of the best signal/blank ratio obtained when detecting fetuin, a model glycoprotein, previously immobilised onto BLI sensors using EDC/sulfo-NHS coupling. Experimental conditions: Fetuin (0, 25 µg.mL^{-1}); SNA@AuNP (OD 1.2). Error = ±S.D, n=2.

Evaluation of SNA@AuNP conjugates reproducibility

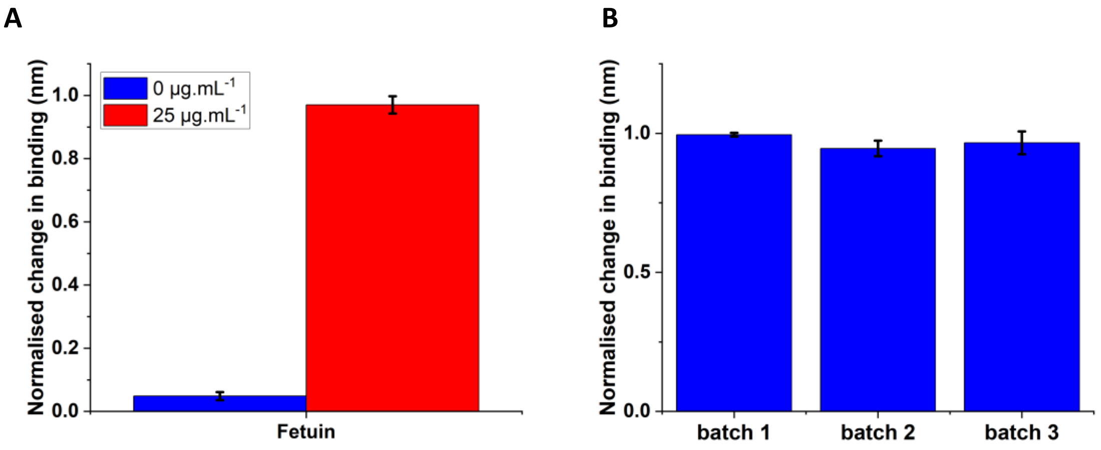

Figure S6. Evaluation of SNA@AuNP conjugates (A) inter- and (B) intra-batch reproducibility. Function determined in BLI by binding to fetuin (model glycoprotein) functionalised BLI sensors. Fetuin was immobilised through EDC/sulfo-NHS coupling and detected with the prepared SNA@AuNP conjugates. Experimental conditions: Fetuin (0, 25 µg.mL^{-1}); SNA@AuNP (coated with SNA 50 µg.mL^{-1}; OD 1.2). Error = ±S.D, n=3 (A) and n=2 (B) per batch.

Stability study of SNA@AuNP

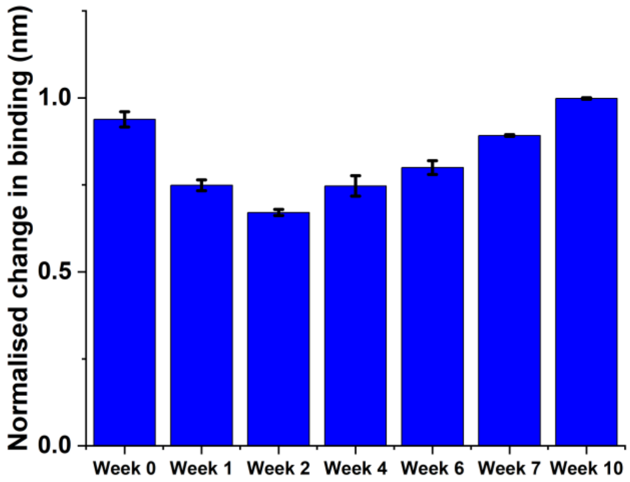

Figure S7. Evaluation of the long-term stability of SNA@AuNP after production and storage at 4°C, over a 10-week period. Function determined in BLI by binding to fetuin functionalised BLI sensors. Fetuin was immobilised through EDC/sulfo-NHS coupling and detected with the prepared SNA@AuNP conjugates. Experimental conditions: Fetuin (25 µg.mL^{-1}); SNA@AuNP (coated with SNA 50 µg.mL^{-1}; OD 1.2). Error = ±S.D, n=2.

Evaluation of the non-specific interactions for different Lectin@AuNP conjugates in the presence of PSA

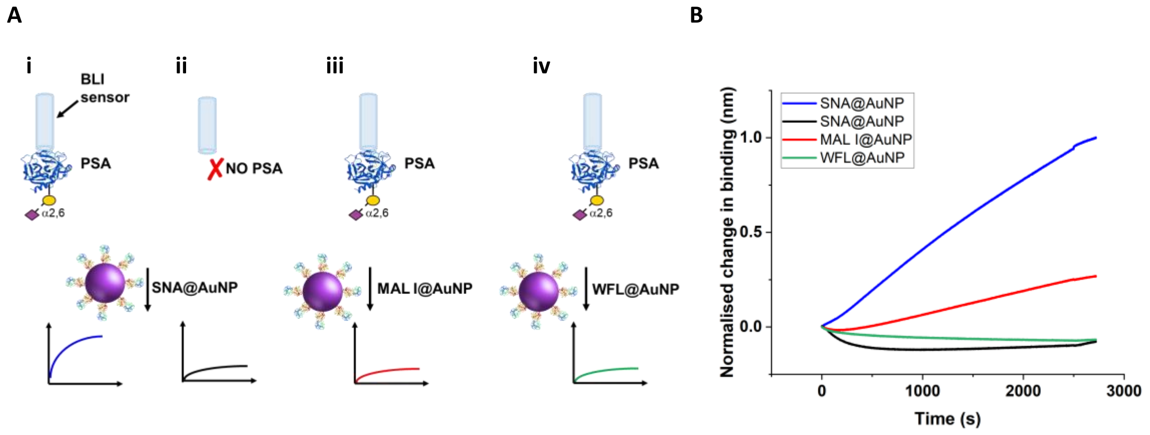

Figure S8. A) Schematic of PSA (i, iii, iv) captured to a BLI sensor using EDC/sulfo-NHS coupling ((ii) as negative control) and B) obtained signal following interrogation with SNA@AuNP (i, ii), MAL I@AuNP (iii) and WFL@AuNP (iv) conjugates. Experimental conditions: PSA (0 (ii) and 0.4 μg.mL^{-1} (i, iii, iv)); Lectin@AuNP (OD 1.2) used were all coated with 50 μg.mL^{-1} of each lectin tested.

Optimisation of capture antibody (anti-PSA antibody) loaded on sensor tip

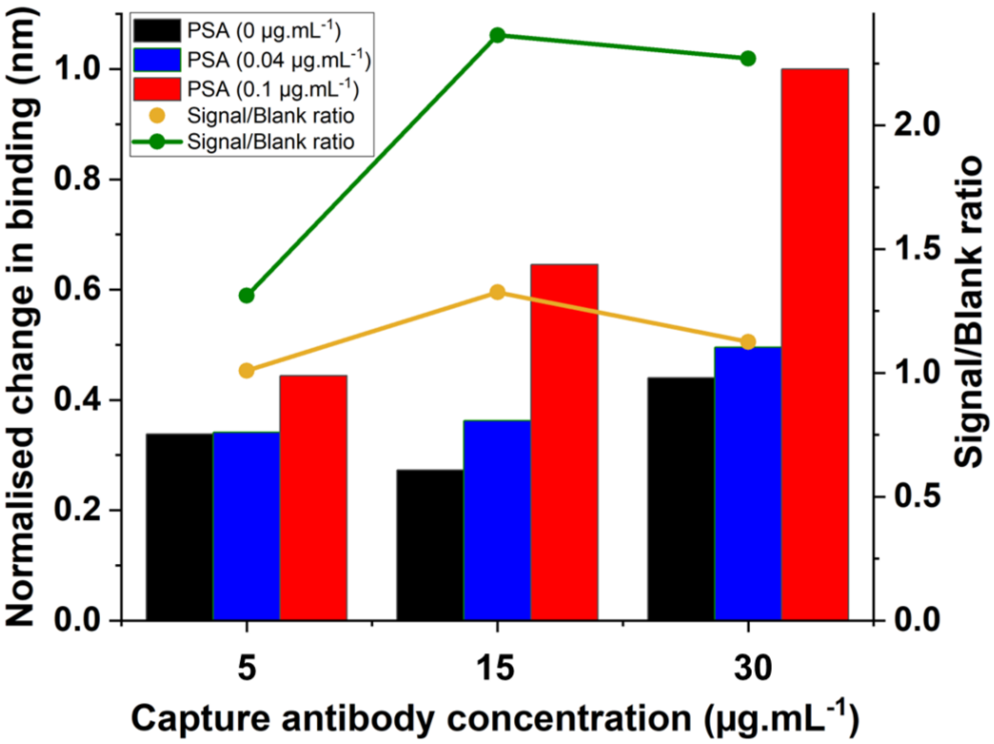

Figure S9. Optimisation of capture antibody concentration immobilised onto the BLI sensor tip using EDC/sulfo-NHS coupling. The optimum amount of antibody was evaluated in terms of the best signal/blank ratio obtained when measuring three PSA concentrations, captured by the antibody, and detected by SNA@AuNP conjugates. Experimental conditions: PSA (0, 0.04, 0.1 µg.mL^{-1}); SNA@AuNP (coated with SNA 50 µg.mL^{-1}; OD 1.2)

Determination of PSA in spiked serum samples

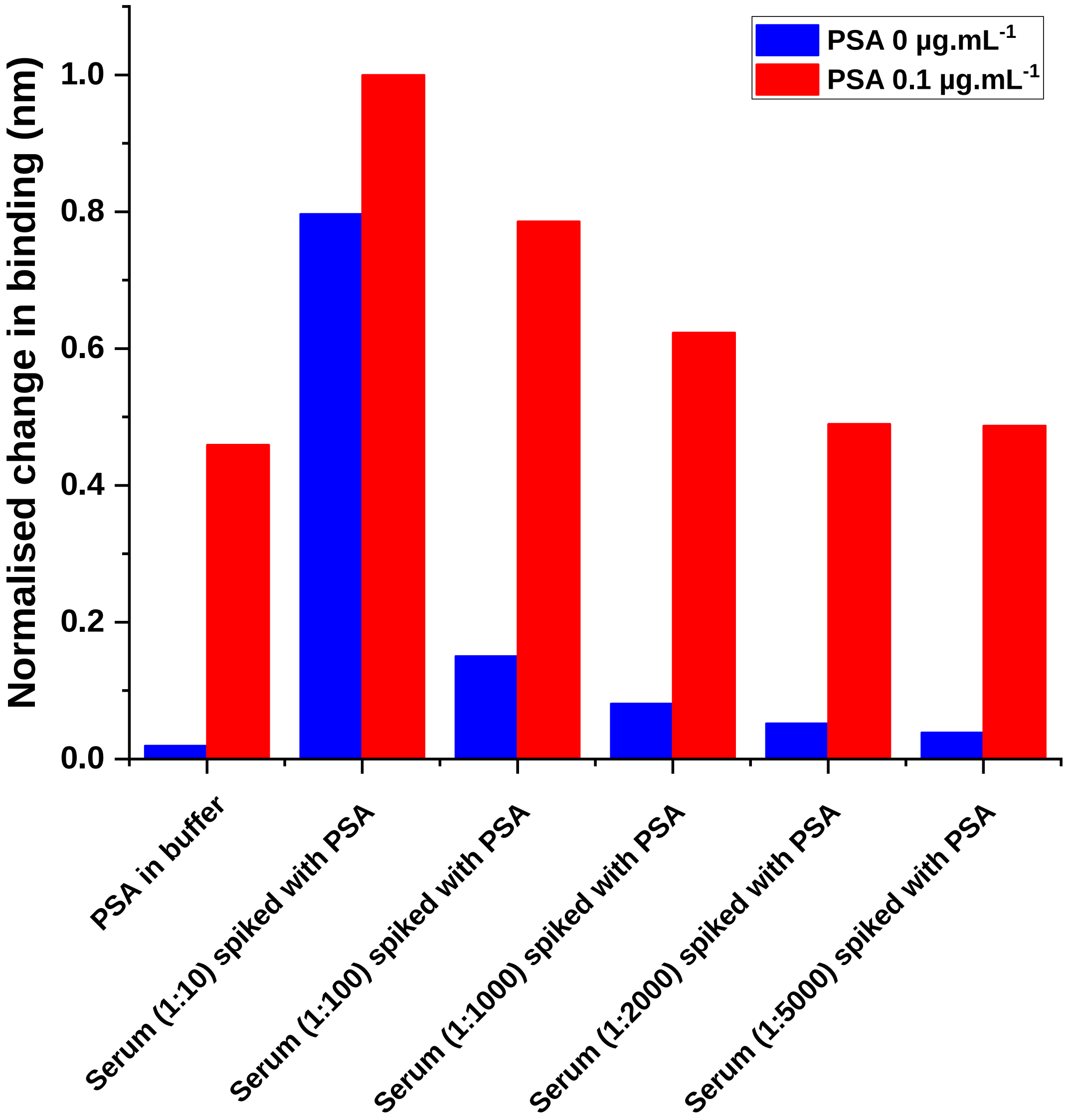

Figure S10. Preliminary evaluation of matrix effects in serum spiked with PSA (n =1). Experimental conditions: Capture antibody (15 µg.mL^{-1}); PSA (0, 0.1 µg.mL^{-1}); SNA@AuNP (coated with SNA 50 µg.mL^{-1}; OD 1.2).

References

(1) Bastús, N. G.; Comenge, J.; Puntes, V. Kinetically Controlled Seeded Growth Synthesis of Citrate-Stabilized Gold Nanoparticles of up to 200 Nm: Size Focusing versus Ostwald Ripening. *Langmuir* **2011**, *27* (17), 11098–11105.
